# Molecular docking analysis of phytocompounds from Andrographis paniculata binding with proteins in the notch-signaling pathway

**DOI:** 10.6026/97320630016923

**Published:** 2020-11-30

**Authors:** Sumetha Suga Deiva Suga, Surapaneni Krishna Mohan, Radhika Nalinakumari Sreekandan, Malathi K, Devakumar Kamaraj, Vishnu Priya Veeraraghavan, Selvaraj Jayaraman, Ponnulakshmi Rajagopal

**Affiliations:** 1 Department of Microbiology, Panimalar Medical College Hospital & Research Institute, Varadharajapuram, Poonamallee, Chennai - 600 123, India; 2 Department of Biochemistry and Department of Clinical Skills & Simulation, Panimalar Medical College Hospital & Research Institute, Varadharajapuram, Poonamallee, Chennai - 600 123, India; 3 Department of Clinical Skills & Simulation, Panimalar Medical College Hospital & Research Institute, Varadharajapuram, Poonamallee, Chennai - 600 123, India; 4 Department of Biochemistry and Department of Research, Panimalar Medical College Hospital & Research Institute, Varadharajapuram, Poonamallee, Chennai - 600 123, India; 5 Department of Pharmacology, Panimalar Medical College Hospital & Research Institute, Varadharajapuram, Poonamallee, Chennai - 600 123, India; 6 Department of Biochemistry, Saveetha Dental College and Hospitals, Saveetha Institute of Medical and Technical Sciences, Saveetha University, Velappanchavadi, Chennai-600 077, India; 7 Central Research Laboratory, Meenakshi Academy of Higher Education and Research (Deemed to be University), West K.K. Nagar, Chennai-600 078, India

**Keywords:** Gastric Cancer, Notch signaling pathway, Andrographis paniculata, Molecular docking studies

## Abstract

It is of interest to document the molecular docking analysis of phytocompounds from Andrographis paniculata binding with protein NOTCH1 in the Notch-signaling pathway in the context of cancer. Hence, we document the binding features of neoandrographolide, 14-deoxyandrographolide, androgapholide and andrograpanin with proteins in the notch-signaling pathway for further consideration.

## Background

It remains one of the most dangerous malignancies and the second largest cause of cancer-related deaths in the world, considering the increasing the occurrence of gastric carcinoma [1,2]. According to epidemiological reports, infections with Helicobacter pylori, diet, environment, host genotype, and smoking are among the causes of gastric carcinoma [3,4]. A large number of studies have shown that irregular signalling pathway expression can play a direct or indirect role in tumor-related gene regulation and promote gastric cancer through complex processes and interactions [5]. Notch signalling is an evolutionarily maintained signalling cascade that regulates several cellular processes, like determination of cell fate, cell division, proliferation, tumour angiogenesis, preservation of stemness and apoptosis, regulated by cell-to - cell communication and crosstalk with other signalling pathways [6]. The Notch families are transmembrane proteins that act to control membrane proteins and transcriptional agents of nuclear energy. Four Notch receptors (Notch1-Notch4) and five DSL ligands (Jagged1, Jagged2, Dll1, Dll3, and Dll4) have been shown to exist in mammals. Notch signalling is activated using the binding the ligand and the receptor in the adjacent cells. The Notch intracellular domain (NICD) is released into the cytoplasm after two consecutive proteolytic cleavages, mediated by ADAM / TACE at the extracellular domain and the γ-secretase complex at the transmembrane region. It then translocates into the cytoblast and replaces a co-repressor complex with the transcriptional repressor C-promoter binding factor-1 (human CBF1 also referred to as CSL). Finally, in the He s and Hey subfamilies, the CSL complex targets and activates the genes of effectors such as genes [7,8]. In addition, in gastric cancer tissues, the Notch pathway effectors are highly expressed compared to adjacent normal gastric epithelium and is associated with poor patient prognosis [9]. It is documented that Notch, STAT3 and Twist signalling interactions in gastric carcinoma have a significant role in promoting the growth of gastric cancer. In gastric adenocarcinoma SC-M1, HEK293 and K562 cells, Notch1 activation increases Twist expression and phosphorylated STAT3 levels. In addition, over-expression of the intracellular domain of the Notch1 receptor (N1IC) enhances the development of gastric cancer, including tumour formation, metastasis, migration and invasion, by facilitating the intercellular interplay of STAT3 and the promoter of Twist [10]. Attention to Andrographis paniculata Nees, a well-known plant from the traditional Indian and Chinese medicinal systems is gaining [11]. Recent pharmacological trials have indicated that AP has both in vitro and in vivo anti-tumor and immunomodulatory effects [12]. It was responsible for its activities for secondary metabolites including diterpenes, lactones and flavonoids occurring in AP [13]. Therefore, it is of interest to document the molecular docking analysis of phytocompounds from Andrographis paniculata binding with protein (NOTCH1) in the Notch-signaling pathway in the context of cancer.

## Materials and Methods:

### Protein Preparation:

The structures of the target receptor of human Notch-1 (PDB: 2VJ3), was downloaded from protein databank. Before using this structure for docking studies, the heteratoms, water molecules and co-crystallized ligands were removed and then it was saved as.pdbqt format.

### Ligand Preparation:

Ten Andrographis paniculata chemical components have been obtained from the Pubchem database (Table 1 - see PDF). In this analysis, we used pdb coordinates for all hydrogen output formats. The charges were further repaired by inserting partial gasteiger charges and then push the autodock. Then the structure of the compounds was opened on PyRx by clicking on Load Molecule and making ligand.

### Molecular docking studies:

Many docking algorithms becomes capable of constructing a wide range of possible structures, so they still need a means to score each structure to categories those of greatest interest. In the present study, the docking process was carried using PyRx 0.8 with the Autodock Vina method using the Lamrkian genetic algorithm as the score function was completed [14,15]. Possession of the ligands located on the basis of the highest binding energy. The PyMol molecular viewer (http:/www.pymol.org/) was used for the study of docked structures.

## Results and Discussion:

**Figure 1:**
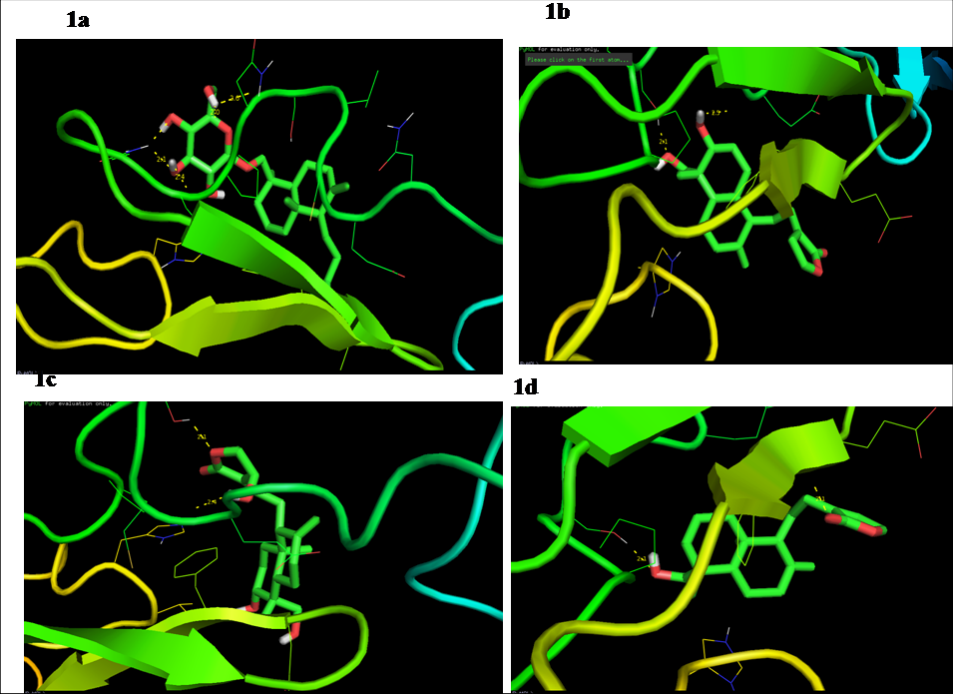
Molecular interaction of Notch 1 with (a) neoandrographolide; (b) 14-deoxyandrographolide; (c) androgapholide; (d) Andrograpanin. Yellow Color dotted line indicates the hydrogen bond interaction

Molecular Docking is an important tool for predicting ligand modes with the protein of known three-dimensional structures. In order to elucidate key structural characteristics and interactions, studies on binding modes are important and provide helpful evidence to develop successful inhibitors. Protein molecular docking experiments, specifically the Notch-1 protein with active Andrographis paniculata compounds was carried out in the current study. (Table 2 - see PDF) displays the docking results. Examination of the results shows all the compounds have greater affinity for the preferred target protein Notch-1. These compounds can also be used successfully to control the Notch signaling pathway. The best four compounds (Neoandrographolide, Andrograpanin, Androgapholide and 14-deoxyandrographolide) were chosen based on these binding affinities. The main contributors to the receptor protein's stability tend to be hydrogen interactions. Thus, in current study, docking experiments have shown that interactions with hydrogen bonding have been mediated in each ligand-protein interaction by particular amino acid residues. In specific, for most compounds, the amino acids SER-458, GLU-455, PHE-474, ASN-459, PRO-460 & GLN-462 alternately form the H bond. Four compounds had been picked out of 10 compounds and shown in (Table 2 - see PDF). Analyzing the docking outcome shows that several of the compounds exhibited score-based and interaction-based interaction with the target protein, and we observed that although these four compounds exhibited very strong activity against Notch-1 protein. Neoandrographolide has found a very high energy binding value (6.1 kcal / mol) across all the compounds, suggesting that it can bind successfully to Notch-1. As described above, neoandrographolide was observed to have the largest number of interactions. It formed the four hydrogen bond interaction via the amino acid residues SER-458, ASN-459, PRO-460 & GLN-462 at distances of 2.6, 2.0,2.4 & 2.1 respectively (Figure 1a). The two hydrogen bond interactions with Notch-1 by SER-458 & GLU-455 were formed with the compounds of 14-deoxyandrographolide and Androgapholide (Figure 1b & c). The hydrogen bond distance was also less than three, with the close interaction with Notch-1 being shown by both compounds. Two hydrogen bond interactions were formed with Andrograpanin through SER-458 & PHE-474 at distances 2.1 & 2.1 (Figure 1d). In all cases, SER-458 Notch-1 residues were known to be involved in the bonding of hydrogen. These amino acids can play a primary role in the protein's function. This has verified that these phytocompounds in gastric cancer can function as a potent inhibitor of Notch-1 protein.

## Conclusion

We document the molecular binding features of neoandrographolide, 14-deoxyandrographolide, androgapholide and andrograpanin from Andrographis paniculata with proteins in the notch-signaling pathway for further consideration.
